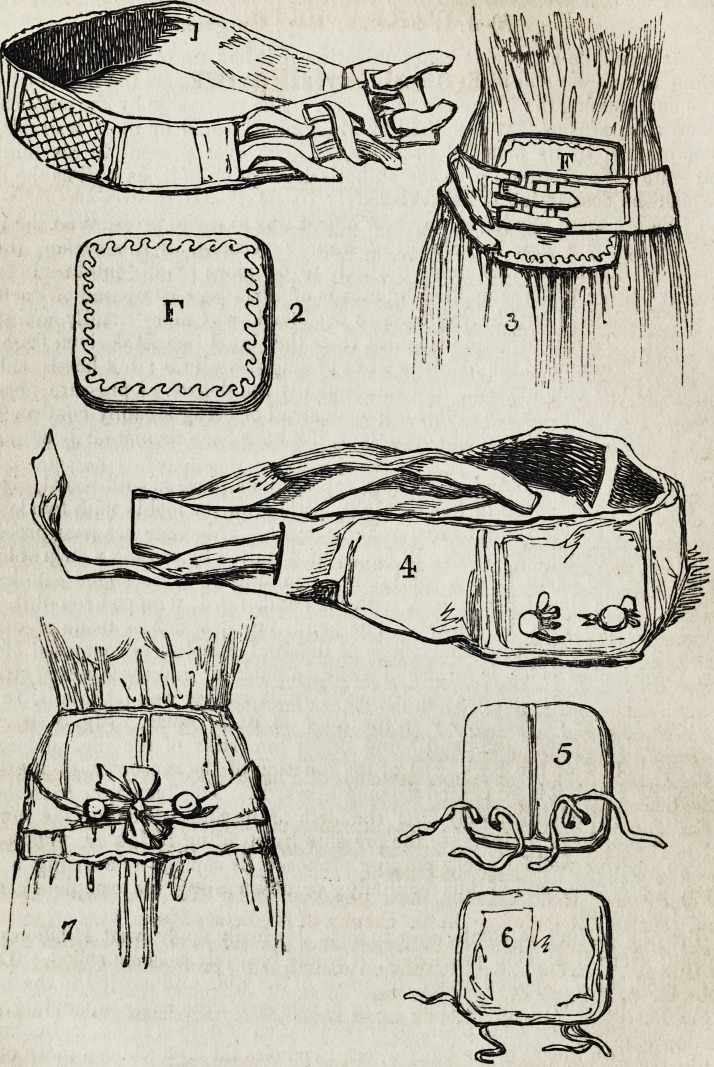# The British Journals: Midwifery

**Published:** 1838-10

**Authors:** 


### MIDWIFERY.

### *On the best Means of applying Pressure to the Uterus after Delivery.*
By J. L. FENNER, Esq., Surgeon.

[THIS would be a valuable communication if it had no other merit than that of tending to impress still more strongly on young practitioners the universal necessity of compressing the uterus after delivery. The possession by the accoucheur of such an apparatus as that described by Mr. Fenner would be most desirable, if it served merely, by its material presence, to keep ever present to his mind that great truth. We therefore give the author's description of it, as well as the figures by which the description is illustrated.]

IN forming these bandages, my first object was to make pressure on the region of the uterus by a *firm unyielding substance;* because, by this means, the contraction of that organ was found not only to be more readily produced than by a similar degree of force applied by means of a bandage composed of linen or of any soft substance, but, having been so produced, was more readily maintained contracted. To this principle we must refer the signal benefit derived from pressure by the firmness of the hands in cases of sluggish uterus: but hands and arms soon tire at this employment, and consequently the degree of pressure necessary to the complete contraction of this organ, instead of being steadily continued, becomes relaxed, and hemorrhage occurs, or, if it have been momentarily suspended, is renewed.

Figure 2, in the subjoined engraving, represents a piece of mill-board, obtained from the stationer, seven inches by eight, padded on the inside with two layers of wadding, and covered with flannel or kerseymere. This plate has been previously divided down the middle, as seen in fig. 5; then united by pasting a strip of leather on each side, so as to form a joint; thus enabling it to be folded into half its compass, like a closed book; and, with the band, fig. 1, wrapped round it, to be conveniently put into the pocket. The band, fig. 1, which is made of variable length, to suit the different dimensions of different females, is composed of webbing, three inches wide; is furnished with two buckles, and three sets of straps to regulate its pressure; and has four inches of India-rubber web let into it, so as to combine a degree of elasticity with the force of its pressure. Fig. 3 shows the bandage duly applied; the band being under the crests of the ilia, and carried round the hollow of the back, just at the junction of the sacrum with the spinal column, by which it is prevented slipping upwards. This bandage, from its easy application, I use immediately after the birth of the child, directing the nurse, if there be hemorrhage, to increase the pressure by buckling it tighter. This simple bandage answers well for every purpose proposed, is capable of exerting a great degree of pressure, and of thus facilitating or accelerating the complete contraction of the uterus.

When the patient is comfortably in bed, I usually apply what I call my sash bandage: were it applied previously, it would probably become soiled. This is represented, fig. 7, applied under the crests of the ilia, and carried to the hollow of the back, just above the sacrum. Fig. 5 represents the exterior, and fig. 6 the interior, of exactly the same plate, with the joint as described in fig. 2; but on each side, within two inches of the bottom, are two holes through which a piece of tape is seen passed from the inside, to attach a pearl button on the outside, of the size of half-a-crown, as seen in fig. 4. Fig. 4 shows this bandage before it is applied, folded in half: it is about thirty inches long, and made of white jean doubled; it incloses the plate, fig. 2; it tapers from the width of the plate towards each end, where twelve inches of strong broad tape are attached for tying under the button, as seen in fig. 7.

The bandage is sloped downwards to fit the hollow above the sacrum, and in its posterior portion a slit is made, through which its opposite end is passed. By placing the plate over the region of the uterus, carrying the two ends of the bandage to the hollow above the sacrum, and then bringing them round under the crests of the ilia, drawing them tightly over the plate, and tying the tapes in a firm manner under the buttons, a very effectual resistance is offered to the tendency which otherwise every bandage would have to slip upwards, and recede from the part which should receive pressure.

The specific advantage of the above plan, besides affording an extraordinary degree of comfort to the patient by the support it affords to the relaxed abdominal parietes, thus preserving the natural figure, is found by experience to be the prevention of uterine hemorrhage and its dreadful consequences. Under the pressure which this bandage is capable of producing, even the formation of a coagulum of any size is almost impossible; and thus the accoucheur is enabled to leave his patient in a state of perfect security, which never can be the case if the uterus, though contracted at the time, be left without the support of some such pressure, which is therefore essential in every case in a greater or less degree. The natural expulsion of the placenta will be much accelerated by the systematic pressure.

*Lancet. June* 2, 1838.

## Figures and Tables

**Figure f1:**